# The Multilayer Network Approach in the Study of Personality Neuroscience

**DOI:** 10.3390/brainsci10120915

**Published:** 2020-11-27

**Authors:** Dora Brooks, Hanneke E. Hulst, Leon de Bruin, Gerrit Glas, Jeroen J. G. Geurts, Linda Douw

**Affiliations:** 1 Department of Anatomy and Neurosciences, Amsterdam University Medical Centres, Vrije Universiteit Amsterdam, Amsterdam Neuroscience, 1081 HZ Amsterdam, The Netherlands; he1.hulst@amsterdamumc.nl (H.E.H.); j.geurts@amsterdamumc.nl (J.J.G.G.); l.douw@amsterdamumc.nl (L.D.); 2 Department of Philosophy, VU University, 1081 HV Amsterdam, The Netherlands; lcdebruin@gmail.com (L.d.B.); glasg@xs4all.nl (G.G.); 3 Department of Philosophy, Radboud University, 6525 HT Nijmegen, The Netherlands; 4 Department of Radiology, Athinoula A. Martinos Center for Biomedical Imaging, Massachusetts General Hospital, Boston, MA 02129, USA

**Keywords:** personality neuroscience, individual differences, network neuroscience, multilayer network, five-factor model, symptom network

## Abstract

It has long been understood that a multitude of biological systems, from genetics, to brain networks, to psychological factors, all play a role in personality. Understanding how these systems interact with each other to form both relatively stable patterns of behaviour, cognition and emotion, but also vast individual differences and psychiatric disorders, however, requires new methodological insight. This article explores a way in which to integrate multiple levels of personality simultaneously, with particular focus on its neural and psychological constituents. It does so first by reviewing the current methodology of studies used to relate the two levels, where psychological traits, often defined with a latent variable model are used as higher-level concepts to identify the neural correlates of personality (NCPs). This is known as a top-down approach, which though useful in revealing correlations, is not able to include the fine-grained interactions that occur at both levels. As an alternative, we discuss the use of a novel complex system approach known as a multilayer network, a technique that has recently proved successful in revealing veracious interactions between networks at more than one level. The benefits of the multilayer approach to the study of personality neuroscience follow from its well-founded theoretical basis in network science. Its predictive and descriptive power may surpass that of statistical top-down and latent variable models alone, potentially allowing the discernment of more complete descriptions of individual differences, and psychiatric and neurological changes that accompany disease. Though in its infancy, and subject to a number of methodological unknowns, we argue that the multilayer network approach may contribute to an understanding of personality as a complex system comprised of interrelated psychological and neural features.

## 1. Personality as a Complex System

Broadly speaking, the theory of emergence states that behaviour arises as a property of an organised system, where the parts of these systems alone do not give rise to the behavioural properties [1,2]. From genetics to molecular pathways, brain circuitry to environmental and social interactions, it can be hypothesised that multiple systems contribute to the emergence of unique human personalities in much the same way.

Current efforts to describe personality in psychology and neuroscience are predominantly carried out either at the level of individual systems, such as the behavioural or neural level alone, or through top-down approaches which link characteristics of one system, such as aggression, to characteristics of another, such as volume or activity of a brain region. More recently, top-down approaches have been used in combination with brain network measures [3,4], which unlike simple morphological studies which measure localised activity or volume alone, are capable of discerning complex structural and functional patterns across the whole neural level through incorporating interactions between multiple brain regions [5,6].

As we demonstrate using the example of the five-factor model (FFM), top-down approaches have consistently established a link between the neural and psychological levels of personality, indicating that there could indeed be neural components that contribute to the emergence of psychological characteristics. These methods, however, are still vastly constrained by a number of methodological simplifications which may be preventing the complex mechanisms of personality that exist across multiple levels, from being derived. These constraints predominantly rest upon (1) the latent variable model used to define personality traits at the psychological level whereby variation in psychological characteristics are explained by an underlying trait, and (2) the use of latent traits to identify the neural correlates of personality (NCPs) through what is known as a “top-down” approach. This is where a top-down approach infers that the NCPs may explain variation in the psychologically defined trait.

Recently, a body of research has advocated for the use of a network model of psychological characteristics, known as a symptom network, as an alternative to the latent variable approach [7,8]. Through this lens, psychological characteristics are seen in relation to each other and not in relation to a common cause. Using the analogy of aggression to compare the two approaches, a latent variable model would argue that an aggressive trait is causing behaviours such as hostility, being easily provoked, and being physically violent, whereas a symptom network model may find that feelings of hostility are strongly related to being easily provoked, which may in turn be linked to physically violent responses. In this way, aggressive behaviour could be seen as an emergent property of a symptom network composed of hostility, being easily provoked and being physically violent.

Unlike the top-down approach frequently used with the latent variable model, there is currently no defined manner by which to connect networked psychological characteristics of personality, at one level, to the networked characteristics of the brain, at another.

In order to explore this possibility, we discuss a number of novel approaches that allow multiple levels of networked characteristics to be connected, these approaches are broadly known as multilayer networks [9]. These methodologies are not only able to incorporate complexity through networking psychological and neural characteristics, but also allow the application of a variety of network analytical tools capable of deriving potentially relevant patterns that exist between the levels. Such an approach reduces the impact of a-priori statistical simplifications intrinsic to top-down and latent-variable approaches. If successful, the multilayer approach could precipitate a formulation of personality that more veraciously describes and predicts individual differences and changes that accompany psychiatric and neurological disease. Through exploring a preliminary methodology for such an approach, we find a strong basis for the idea that the study of a complex, emergent system such as personality, including neural and behavioural, cognitive and emotional characteristics (BCECs), would benefit from an integrated, multilayer network approach.

## 2. The Study of Personality

Current models in psychology often conceptualise personality as a series of ‘relatively enduring’ behavioural, cognitive and emotional characteristics [10]. The psychology of personality often focuses on explaining the variability in these characteristics as a result of variability in an underlying, or ‘latent’ trait [11]. This methodology is known as a latent variable model. Since traits cannot be directly measured, statistical approaches such as principle component analysis and independent component analysis are used to derive traits that best explain the variability in the observed BCEC. Using the widely accepted five-factor model (FFM), it is argued that the observed variance in 240 characteristics assessed by the NEO Personality Inventory (NEO-PI-R) self-report questionnaire, is explained by five core traits: neuroticism, extraversion, openness to experience, agreeableness, and conscientiousness and 30 sub traits known as facets [12]. For example, it would be expected that a high score in the assessed characteristic of making friends easily could be explained by the trait extraversion, and the facet friendliness.

On the basis that BCECs emerge from specialised mechanisms in the brain responding to their environment, personality neuroscience, by contrast, is concerned with determining a neurobiological basis to variation in BCEC. In order to do so, personality neuroscience typically follows one main approach, whereby scores in psychologically derived traits, such as extraversion, are correlated to variation in brain characteristics, such as grey matter volume or brain network characteristics. This is known as a top-down approach and infers that a personality trait or facet is explained by variation in an underlying brain characteristic. For clarity, these brain characteristics will be termed the neural correlates of personality (NCPs). The definition of NCPs has increased in acuity through technological advances, progressing from morphological studies in post-mortem to the use of magnetic resonance imaging (MRI) [13,14,15,16] and surfaced-based morphometry indices [17]. Functionally, task-based brain activation as assessed with functional magnetic resonance imaging (fMRI) has been explored. More recently, however, the field has been directed by studies which use structural connectivity measured with diffusion MRI, but also the functional connectivity measures derived from resting state electroencephalography (EEG), magnetoelectroencephalography (MEG) and resting state fMRI (rsfMRI) [18]. Over recent years, a multitude of these novel tools have been used to investigate the origins of the BCEC of personality in the brain with top-down approaches, where the psychologically defined traits of the FFM are most frequently used as higher-level concepts.

### Simple Morphological Studies

**Figure 1:**
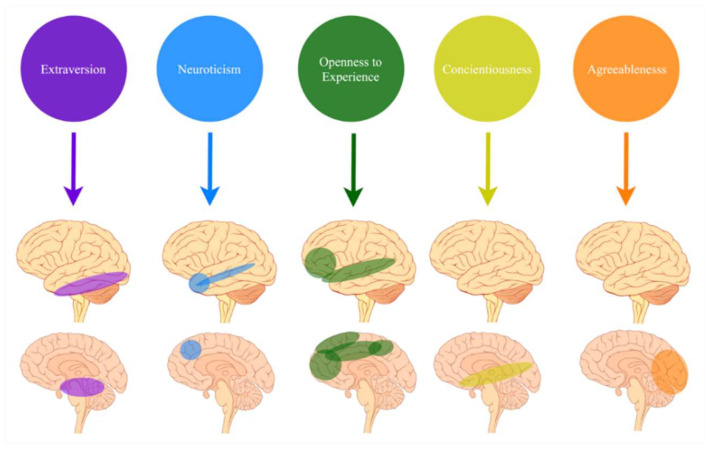
Schematic demonstration of the top down approach to personality study. Arrows in this figure represent the direction by which lower level concepts, in this case brain connectivity [21], are defined through use of the higher-level concepts of the FFM trait measure. The direction of this definition means that brain regions and/or connectivity are dependent upon the higher-level concept of the personality trait.

The use of the FFM in top-down simple morphological studies has demonstrated one-to-one correlations between varying scores in a personality trait, and differences in the anatomical features and activation of localised brain regions (Figure 1). For example, research has indicated that grey matter volume [14,15,16], white matter integrity [13,19] and surface-based morphometry indices [17] are related, though not always consistently, to the FFM trait scores. The inconsistencies are often attributed to the use of small sample sizes, of approximately 100–200 participants, and heterogeneous samples that often include individuals from single cultures or locations [20].

These approaches have precipitated much of the current understanding about the relationship between the neurological characteristics and BCEC of personality [13,14,15,16,17]. However, it cannot be overlooked that this is an inherently top-down approach; meaning that the features of personality are explained by correlations to circuits or regions in the brain. By isolating and attempting to pinpoint single brain characteristics, such as regional grey matter volume, white matter integrity or surface folding, simple morphological studies are not able to include interactions in the larger system, such as: (1) volume and activity of brain regions both impacting one-another and BCECs, and (2) BCECs both impacting one-another and the brain.

This means that simple morphological studies may be overlooking factors such as complexity and detail at the neural level [3], but also at the level of BCECs due to the use of a latent variable model [11]. This is certainly not an issue confined to the study of personality neuroscience, as simple morphological studies are arguably the basis of any biological study of structure-function relationships, however, with the advent of new technologies and approaches in the field of complex systems, there is the possibility to incorporate a greater degree of multimodal and interrelated data to the study of structure-function relationships such as that between the brain and BCECs. One such way is a network approach.

## 3. Complex Networks

Network science is an emerging field of study which aims to model complex natural phenomena as dynamic and interactive systems (Table 1).

Network science is an extension of graph theory, differing in the sense that components within a model are given attributes, as opposed to representing arbitrary objects. Formally, an undirected network would be defined as G_A_ = (V_A_, E_A_), where V_A_ refers to the set of nodes and E_A_ the set of edges defined as ((u, α), (v, α)) ∈ E_A_ if (u, α), (v, α) ∈ V_A_. In practice, this generally entails modelling real world interactions, known as ‘edges’, between phenomena, known as ‘nodes’, as a means to understand how both the characteristics of individual components and the whole interactive system could give rise to system behaviour.

In network science, individual separable components (the nodes) require definition by observation and study as they are not necessarily ‘separate’ phenomena in their natural state. In network neuroscience for instance, these nodes are usually defined as the regions of the brain with separable cytoarchitecture, structural connectivity and/or functional connectivity, analogous to stations being nodes in the railway system.

Taken together, a network approach thus allows for incorporation of several characteristics of phenomena and the interactions between them, generating a greater degree of complexity. Furthermore, network analyses can be applied in order to assess and interpret the characteristics of the network. These include: node degree and strength, clustering, centrality, modularity and efficiency (see Table 1 for detailed definitions). Over the past decade, network and graph approaches have continued to demonstrate success in modelling multiple complex systems, including biological, technological and social systems, but more recently in understanding complex interactions at the level of the brain [22] and the level of psychology [23,24].

### 3.1. Network Neuroscience Techniques

Network neuroscience [22] or the study of the brain network, has been on the rise since the introduction of tools that allow the measurement of structural and functional connectivity. Structural connectivity refers to the anatomical connections between brain regions. Such connections are most often measured using diffusion weighted MRI techniques which characterise white matter pathways and may map the orientation and integrity of white matter fibre bundles [25]. Along with drawing conclusions about structural connectivity between regions, this structural connectivity data can be analysed in terms of its network properties, allowing conclusions to be drawn about, for example, the efficiency of the structural connections throughout the whole brain. Diffusion weighted MRI, as a measure of structural connectivity, has been explored with regards to the five-factor model of personality [26]. In that study white-matter integrity was implicated in mediating neuroticism and openness-to-experience, though in this instance the properties of the structural network as distinct from structural connectivity, were not considered.

Functional connectivity, by contrast, is concerned with modelling connections between brain regions based on their co-activation. For example, if two regions are active simultaneously, they are said to be functionally connected. A weight can also be given to the functional connection dependent on the strength of the signals. Resting state fMRI (rsfMRI) is the most common means to measure functional connectivity and involves quantifying the flow of oxygenated blood to brain regions while a participant is not engaged in activity. The use of rsfMRI has prompted an understanding of the more stable states of functional connectivity that occur in the resting brain and have allowed for a number of large-scale networks to be identified using resting state functional connectivity data. These include the well-established default mode network, the limbic network and the salience network [27] and have to varying degrees been related to a number of BCECs of personality.

For example, the default mode network is a pattern of brain connectivity that activates when an individual is at rest, and is comprised of three main sub-divisions: the medial prefrontal cortex, the posterior cingulate cortex, and the precuneus together with the lateral parietal cortex (Raichle, 2015). Through studies in both humans [28,29,30,31,32], and primates [33], the default mode network has been consistently associated with the motivational, social and affective components of behaviour, which together play important roles in personality. Meanwhile, trait openness-to-experience, which is highly correlated with imagination and intellect, is found to be related to dynamic shifts between a number of large-scale networks including the default mode, salience and executive networks [34]. A concern about rsfMRI, and fMRI in general, however, is that there may be a delay in blood reoxygenation after activation of a brain area, thus the technique may not give a temporally accurate model of brain functional connectivity.

EEG and MEG are two further functional neurophysiolgical techniques that in contrast to rsfMRI have high temporal accuracy, but low spatial accuracy. Respectively, EEG and MEG detect fluctuations in the brain’s electrical and magnetic fields that vary in response to post-synaptic activity [35]. However, the technique requires large numbers of synapses to be active simultaneously to give rise to a detectable signal, hence the low spatial acuity.

Together, the brain network approach, and the vast amount of data that has been collected over the past two decades, now allow network neuroscience to draw novel conclusions on the connections present in complex brain networks and their relationship to the emergence of function. Network measures have shown great promise in progressing understanding throughout a broad range of topics in neuroscience, from neurodevelopment to the neural basis of cognition [5,6]. Its introduction into the study of personality, however, is relatively new.

### 3.2. Personality Network Neuroscience

Over the past decade, attempts were initiated to utilise this interconnected model of the brain in order to search for the NCPs that could underlie human personality [4]. These studies can be categorised into two types: (1) personality connectivity studies which solely investigate connectivity between brain regions and (2) BCEC network studies which analyse the characteristics of the brain networks related to personality. As it stands, there is not a great many studies focusing on the relationship between personality traits and either brain connectivity or brain network characteristics. Furthermore, difficulty often arises in corroborating correlations between studies due to a wide range of models used to define personality on the one hand and the brain network on the other hand. A comprehensive review of these studies is beyond the scope of this article, but the reader may refer to two recent literature reviews [3,4]. Within these reviews, an emerging majority of studies uses the personality trait model FFM in combination with brain network investigation. It is for this reason that the current article focuses its argument on studies which use the FFM. Although the FFM alone does not represent all aspects of personality study, it is relevant for succinctly demonstrating the implications of using a trait model in combination with brain network measures [19].

### 3.3. Top-Down Approaches in Personality Connectivity Studies

Associations between brain connectivity measures and all FFM personality traits have been investigated in studies of functional and structural connectivity [21]. One such study used rsfMRI to search for correlations between functional connectivity and the FFM traits [21]. The study used the precuneus and anterior cingulate gyrus as seed regions; areas thought to be functionally involved with the default mode network as cognitive and affective hubs. From these seed regions distinct functional connectivity patterns were measured and related to each of the five traits. For neuroticism, functional connectivity between the dorsomedial prefrontal cortex, the middle temporal gyrus and temporal pole was associated with individual differences. Together these regions are implicated in emotional regulation and fearful anticipation, consistent with FFM personality models of neuroticism. Extraversion was associated with resting state functional connectivity in the lateral paralimbic regions, specifically in areas thought to be involved with motivation and reward, and in the fusiform gyrus, an area thought to be involved in social attention and facial recognition. Functional connectivity in the midline (posterior cingulate and anterior medial prefrontal cortex), which are regions considered to be hubs of the default mode network were associated with openness to experience; along with areas of the dorsolateral prefrontal cortex implicated in intelligence and creativity. Agreeableness was associated with connectivity of areas regarded as important in social and emotional function, namely the posteromedial extra-striate regions. Lastly, functional connectivity in the medial temporal lobe, a region involved with deliberation and planning, was associated with conscientiousness. This study provides a preliminary insight into the relationship of functional NCPs relating to personality traits in the resting brain.

### 3.4. Top-Down Approaches in Personality Network Studies

Progressing from connectivity studies that focus on interregional connectivity, a study of 818 participants used network techniques to analyse public data from the Human Connectome Project [20]. This was done in order to relate features of resting state functional networks to the FFM traits. Local network indices were calculated, including: nodal strengths defined as the sum of the edge weights attributed to one node, clustering coefficients defined as the interconnectivity of a node’s neighbours and efficiency defined as the inverse of the shortest average path length of a node. Additionally, global network indices were calculated as the average of local network indices. In this instance, no associations were found between global network indices and any of the five personality traits. However, robust associations were found between conscientiousness and local network indices in the left frontoparietal and default mode networks. It was further found that two facets of conscientiousness, dutifulness and achievement, were positively associated with betweenness centrality, a measure of how important a node is within the network, in the default mode network. Although no fully encompassing network description of all five traits was found, the authors conclude this may not necessarily be the result of these traits not being related to the brain network. Two possible solutions they propose, along with type II errors, are that (1) there are non-linear relationships between network NCPs and personality traits, and (2) that in order to understand these more complex relationships new brain network measures are essential.

### 3.5. Limitations of Latent Variable, Top-Down Approaches

The point about non-linearity in top-down connectivity and network studies links back to the aforementioned arguments describing why simple morphological studies may falter in describing mechanisms of personality. Although these network studies incorporate a greater degree of complexity at the level of the brain, they are limited by their dependence on higher-level factors derived with a latent variable model at the level of BCEC. The organisation of the NEO-PI-R facets into traits typically uses statistical approaches, such as principal component analysis (PCA) and independent component analysis (ICA) from observed BCEC. This may lead to a number of simplifications, succinctly summarised in the words of a study [36] that compared the networked versus factor structure of BCEC:

*“Most importantly, the mutual relationships between the items that make up the factors are not explicitly modelled, and hence disregarded. This may be unfortunate, since some of these interactions may have disproportionate importance when compared to others (e.g., some items may be correlated to many or fewer other items, show stronger or weaker correlations, explain more variance in factor scores, or have causal dominance over others). As a result, items, facets and factors of the NEO-PI-R are given the same weighting and diagnostic value. This may not be desirable, given the possibility that certain personality traits (such as neuroticism or agreeableness) have a disproportional importance in mediating healthy personality development.”*

This quote implies that when latent trait variables are used as higher-level concepts in a top-down approach when studying the brain, this simplification at the psychological level may be confounding the biological relevance of the NCPs observed. As with the progression of morphological studies to network studies at the level of the brain, including interactions at the psychological level may allow greater detail and complexity to be observed.

### 3.6. Incorporating Interactions at the Psychological Level

In psychology it has long been observed that psychological characteristics are often accompanied by comorbidities, which appear as if they may have causal influence over one-another. For those that advocate for a network model of psychological characteristics, this observation suggests that psychological disorders or traits are not necessarily causing their symptoms, as in a latent variable model [8].

**Figure 2:**
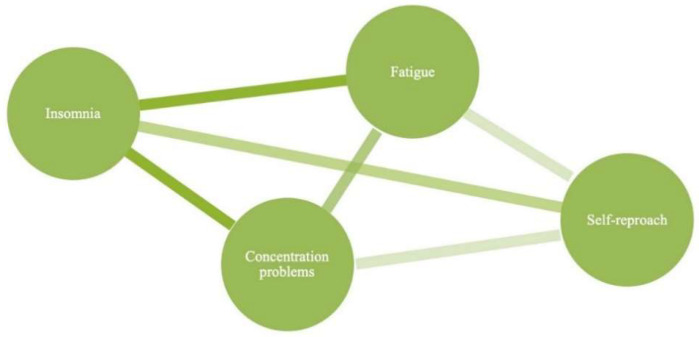
Symptom Network Model of Psychological Features. This figure shows a hypothetical symptom network model of the symptoms: insomnia, fatigue, concentration and self-reproach. The symptoms are connected by edges that could represent causal relationships or co-occurrence. The edges further have weights which represent the magnitude of the relationship between two symptoms, in this example, insomnia and fatigue would be highly related, but self-reproach and concentration less so. Together, the symptom network approach endeavours to view symptoms in relation to each other, which removes the need for a unified underlying cause to higher-level psychological terms.

Instead, that psychological disorders or traits are descriptions, and not causes, of a self-sustaining network of characteristics (Figure 2). A key study on depressive symptoms, for example, showed that “death wishes”, “loss of interest”, “pessimism” and “depressed mood” were highly related symptoms, central to the experience of depression in later life, though not caused by depression itself [37]. In this way, depression could be seen as an emergent property of what is known as a ‘symptom network’. Considering the vast overlap between psychiatric disorders and personality, both being persistent states of BCEC encountered by an individual, the same symptom network approach can be used to model the BCEC of personality [8]. Through this lens, the psychological level of personality would therefore be seen as a unique organisation of networked BCEC, not caused by a latent trait.

Creating a symptom network based on group-level interactions between symptoms involves collecting data about the presence of symptoms within a patient population and investigating how these symptoms are empirically associated [7]. With this data symmetric correlation matrices can be constructed that contain information about symptom-symptom interactions and can then be used to create association networks. These association networks are useful in identifying symptom co-occurrence and clustering, however they do not give information about causal relationships between symptoms, as correlations between symptoms may be the result of an interacting latent variable. For this reason, partial correlation networks, known as concentration graphs, can be used which are thought to provide better insight not only into unidirectional co-occurrence of symptoms, but also directional causal dependencies between symptoms [8,24]. Similar to brain networks, analyses can be used to assess the organisation of the network, including the centrality of certain characteristics, or whether there are diagnostically useful clusters of characteristics.

As it stands, a limited number of studies have used a symptom network model with the BCEC of personality, where one study has investigated the use of the FFM [23]. A method to create BCEC networks is outlined within the study, where similar to psychiatric symptom networks, the nodes are represented by psychological features, in this case the 240 observed characteristics of the NEO PI-R. Edges represent directed and undirected relationships between the characteristics based on group-level correlations [23]. Taken together, this approach offers a model of the BCEC of personality that includes networked interactions, and hence a greater degree of detail and complexity, not included in latent variable models.

Finding a manner by which to utilise the complexity available through the use of networks at both the neurological and psychological levels, is venturing somewhat into a methodological unknown, but a direction with vast potential for new understanding in the field of personality.

## 4. Multilayer Personality Neuroscience

The multilayer network approach refers to a number of methodologies endeavouring to combine two or more networks into a single ‘multilayer’ network [9]. By doing so, the resulting model provides further insight into the organisation and dynamics of a more complete system, potentially superseding the explanatory and predictive power of a single layer network alone.

The many formulations of multilayer networks have sparked interest in fields involved with deriving and predicting complex phenomena, such as biological, social, technical and transport systems [38,39,40,41,42,43]. This interest largely stems from the approach’s capacity to incorporate multiple datasets that involve more than one modality, scale, or point in time, as is often the case in the study of complex systems. A general example of the use of a multilayer network can be seen by expanding the analogy of transport systems:

If one wished to understand why there were frequently traffic jams at the central station of a city, the problem may not be well explained by looking at the cars alone. Instead, an investigation into the interaction between cars, pedestrians, bicycles and trains may (1) yield a more complete model of the system (2) allow for more accurate prediction of the system and (3) allow interventions to be implemented which prevent traffic build-ups and make the system more efficient.

This analogy can be directly translated into the general framework of a multilayer network. Here, the transport modalities are defined as layers, the nodes are defined as geographical locations, the connections between the layers, known as interlayer edges, are defined on the basis that the geographical locations in each layer are identical and can therefore be connected. These identical nodes between layers have been termed ‘replica nodes’ [44]. The presence of replica nodes is fundamental to this formulation of the multilayer network as it allows the different layers to be connected.

Recently, this general formulation of the multilayer network has been used in studies of the brain [45,46,47]. Layers have often been defined on the basis of (1) differing imaging modalities [48], such as one level representing structural connectivity and the other functional connectivity, (2) differing scales within one modality as layers [47] or (3) with time-series data, where layers are represented as different moments in time [49].

This general formulation of the multilayer network which integrates different imaging modalities or time-series data, however, would not be directly applicable to the study of the relationship between brain and behaviour. This is because in these prior examples, interlayer connections are formed on the basis of replica nodes which would not exist across multiple scales such as in the neural and behavioural levels. In this way, the general multilayer method is not able to give an indication of the properties of a system that emerge across the micro and macro levels. The following section discusses a means by which to extend the use of the multilayer network across multiple scales, allowing for the aforementioned benefits to be harnessed in the study of the relationship between the neural and BCEC levels of personality.

### Constructing a Multilayer Network without Replica Nodes

The first step towards creating this multilayer network would involve both brain and BCEC data collection in a sample of individuals. First, for every participant, it would be necessary to collect scores for characteristics in a personality questionnaire such as the NEO PI-R. The normalised scores in each of these characteristics would then be used to create a group level BCEC network (Cramer et al., 2012), using partial correlation networks [24]. This network would represent the BCEC network layer within the multilayer. Second, it would be necessary to measure brain network data for every participant, using for instance rsfMRI. To create the brain network layer, both a normalised group level brain network, or individual level brain networks could be used, where the latter would give a greater indication of heterogeneity between individuals.

**Figure 3:**
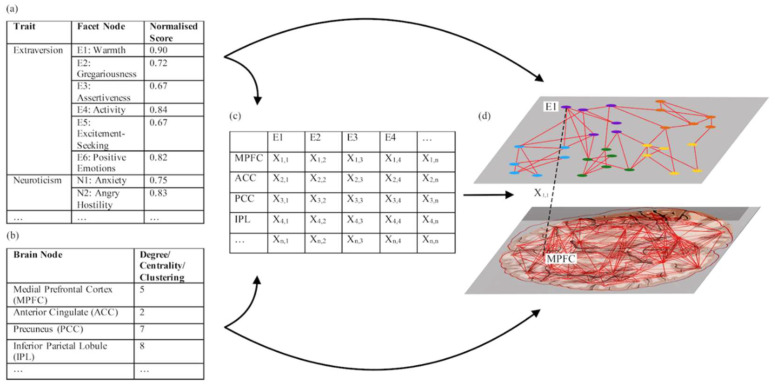
Methodology for multilayer approach without interlayer identities. (**a**) Normalised personality scores in every facet of a personality the FFM (Friendliness = Extraversion One (E1)) are collected from a sample of individuals and used to make a BCEC network by means of the Partial Correlation network methodology. This data is used to create the BCEC network layer in the multilayer network (**d**). (**b**) Brain network nodes are defined and network characteristics, such as degree, clustering and centrality, are quantified for the original sample of individuals. This is used to create a brain network layer in the multilayer network (**d**). (**c**) A matrix is created which quantifies the correlations between each node in the personality data and each node in the brain data, this matrix score is then used to define the weight of the intralayer connections (X_n,n_).

After creating the networks at the level of the brain and BCEC, it is then necessary to relate the normalised mean scores for each node in the BCEC network to each node in the brain network. This is done in order to create weighted interlayer connections, based on correlations between each brain node and each personality node. In order to do this, a quantifiable characteristic of the brain node must be selected, such as the node’s overall connectivity or centrality (Table 1). Both these measures would provide each node with a value, for each participant, within each layer. For example, at the individual level the FFM BCEC network node corresponding to warmth (E1) could have a score of 0.90 (Figure 3a) and the node corresponding to the medial prefrontal cortex (mPFC) could have a score of 5, which could be based on the node’s degree (Figure 3b). Calculating group-level correlations between node pairs is then necessary to quantify the strength of the interlayer connections.

In order to quantify the strength of the relationships between nodes across the two layers, a matrix could be devised using a normal correlation or a non-parametric correlation (Figure 3c). Further research into more rigorous statistical approaches for this step of the methodology is still required and may become clearer as more is learnt about the approach. In theory, however, the outputted matrix scores provided by such a statistical analysis, could then be used as the weighted interlayer connections between the BCEC network and the brain layer (Figure 3d). Together, this would give a multilayer BCEC-brain network (Figure 3d).

This multilayer model would allow network analyses to be carried out over both layers, in order to derive novel interactions between neural and psychological characteristics, and network communities and/or hierarchies that are biologically relevant to the emergence of personality. This may prove useful in first modelling brain-BCEC interactions in personality at a group level, and further in being able to form individual multilayer networks that could provide insight into differences between (sub)groups. An example could be the exploration of hypothesized subtypes of personalities and their possibly distinctive multilayer network topologies. Furthermore, the technique could involve a greater number of layers corresponding to additional networks which could be impacting upon personality trait and brain networks, such as environmental and genetic factors.

Overall, although in its infancy, this multilayer approach allows for personality to be viewed as a dynamic and multifaceted phenomenon, where individual phenotypes can be modelled as an accumulation of variable interactions between multiple layers.

## 5. Discussion

An important limitation of the multilayer approach as a means to model personality follows with the difficulty in defining suitable and representative network nodes in both brain and BCEC networks, even within those systems. At the level of the brain, network nodes, or brain regions, are chosen by the researcher based on predefined notions of the properties of different areas of the brain, be it cytoarchitecturally, structurally or functionally. There is still much debate about the best means to select these regions, as their positioning can have a large impact on the edges that are formed, and thus the topology of the brain network created [50]. Currently anatomical atlases and activation studies are often used as a means to define these nodes, but there is research emerging documenting other means of definition such as the voxel-wise method (Stanley et al., 2013) and using connectivity profiles such as the Brainnetome [51], which could ameliorate some of the problems posed by node definition at the level of the brain.

This limitation of node definition is likewise the case for the selection of nodes in a BCEC network. According to the model proposed by this article the nodes within the BCEC network would be defined on the basis of a personality trait model such as the FFM, and the edges as co-occurrence measured between BCEC. However, it may be the case that the assessed characteristics are not distinct and representative descriptions. We accept that there could be more successful non-trait models of personality that were not considered in this argument, where these models could provide more descriptive nodes in a BCEC network. However, the argument that the latent variable models poses an oversimplification to personality neuroscience regardless of the model used, still holds. Therefore, a final remark on this limitation is that future research is required to investigate the plausibility of the vast number of personality models for definition of descriptive and useful personality nodes within a multilayer network.

Moreover, without extensive temporal information on the interactions between personality traits, the resulting multilayer will always remain at the group-level, not allowing for individualized investigation of brain-behavior interactions.

Furthermore, there are myriad limitations to be overcome in constructing the personality-brain multilayer network. The definition of interlayer connectivity is completely new and hampered by statistical difficulties, as node weights from both brain and behavior may not be normally distributed or even merely ordinal in nature. It is intuitive to associate individual differences in the centrality of a brain node to individual differences in particular facets of personality, but the large number of associations drawn to incorporate all these interactions asks for rigorous statistical methodology that has not been developed yet for multilayer networks with different nodes per layer. Also, it has become clear that differences in connectivity weights and distributions between layers may induce non-existing patterns [46], necessitating some sort of standardization between the layers.

A final possible limitation of our proposal is its assumption that there is a neural component to personality and that it does not exist on a purely behavioural level. We argue that the initial structural and functional studies provide enough evidence to consider a neural element to personality, where a focus on symptom networks in combination with brain networks could be a means to derive further evidence of its organisation.

## 6. Conclusions and Future Directions

The concept of personality is certainly complex, where its various formulations throughout history have individually proven useful in characterising certain enduring behaviours and thus predicting – with varying degrees of accuracy-how similar groups of individuals are likely to behave. From focus on childhood experience to biological models which support the importance of genetic factors, the study of personality throughout the last century has searched for causal phenomena which link these behaviours to measurable origins such as a trauma, brain structure and genetics. However, with the advent of big data in the health sciences, it is becoming possible to model the vast number of phenomena involved in the presentation of BCEC, especially those that do not function on a one-to-one basis as is presupposed in many models of personality.

Multilayer networks offer compelling new frameworks for understanding personality in relation to the brain. By removing the need for higher level concepts, instead modelling personality as a series of layers, multilayer networks allow human individuality to be seen as a complex system of interactions across multiple domains. This may help open a much-needed conversation, not just in the field of personality, but in neuropsychology as a whole, that individuals are vastly variable in many dimensions and variability in these dimensions can cause a number of different expressions. Particularly so, in the sense that individuals with a different accumulation of genetic, brain structural and environmental factors could express similar behaviour and vice versa. As it stands the complexity of current models is unable to capture this difference on multiple scales. Even more so, in terms of understanding brain-behaviour relationships as related to sociocultural factors, the endurance of personality over time and in patterns that could contribute to risk for personality-related diseases.

There is, however, a great deal that still needed to be investigated about the practicalities of multilayer, symptom and brain networks, in terms of defining nodes, layers and interlayer connections. Addressing these methodological considerations fundamentally comes down to the question of determining what measurable or conceptual features of personality are important at each conceptual layer and how they relate to each other. In order to answer this question this article proposes three points of future attention. First, in considering alternative formulations of individual difference apart from trait models that could be used to define the nodes in a BCEC network, second in considering in which ways individual differences relate to each other in order to define edges and interlayer connections, and lastly in developing techniques which allow multilayer models to be informative at both an individual and group level. Together, further research into these domains, and networks as a whole in neuropsychological research, may enhance an understanding of the rich multi-dimensional variation that occurs in every human being.

## Figures and Tables

**Table 1:** Definitions of frequently used network terminology.

Term	Definition
Network	A mathematical framework used to model interactions between objects or phenomena.
Node	An object or phenomenon within a network, for example, a node could be defined as a railway station while modelling a rail network.
Edge	A connection between network nodes, for example, an edge could be defined as the railway track between two railway stations.
Edge-Weight	The strength of the connection between two nodes.
Degree	The number of edges connected to one node.
Directed Network	A network wherein the edges are unidirectional such as from node V_1_ to V_2_, but not node V_2_ to V_1_.
Non-directed Network	A network wherein the edges are bidirectional such as from node V_1_ to V_2_ and node V_2_ to V_1_.
Node Strength	The sum of the edge weights attributed to one node.
Hub	An important node within a network, often defined by its centrality.
Centrality	A measure of node importance, defined in many manners including: degree centrality, closeness centrality and betweenness centrality.
Efficiency	A measure of how well a node is able to communicate with other nodes, either with its neighbours (local efficiency) or with the whole network (global efficiency).
Clustering Coefficient	A measure of interconnectivity of a node’s neighbours (local clustering coefficient) or throughout whole networks (global clustering coefficient).
